# Progress in Wildlife Conservation, Management and Biological Research: From Molecular Perspectives to Ecological Processes

**DOI:** 10.3390/biology15010103

**Published:** 2026-01-05

**Authors:** Ana Carolina Srbek-Araujo

**Affiliations:** Laboratório de Ecologia e Conservação de Biodiversidade (LECBio), Programa de Pós-Graduação em Ciência Animal (PPGCA), Universidade Vila Velha (UVV), Vila Velha 29102-920, ES, Brazil; srbekaraujo@hotmail.com

Wildlife conservation and the management of biological resources face unprecedented challenges in the Anthropocene. Biodiversity loss, driven by habitat fragmentation and degradation, pollution and climate change, for example, demands a robust and multifaceted scientific response. The following Special Issue of *Biology*, entitled “Progress in Wildlife Conservation, Management and Biological Research”, features 11 papers that collectively illustrate the forefront of research applied to biodiversity conservation and management. Together, these studies demonstrate how the integration of advanced molecular methodologies, long-term monitoring and ecological theory is essential for understanding current challenges and for establishing effective actions to protect species and ecosystems.

The published papers reflect an interdisciplinary effort in which biological research supports the identification of threats and generates essential tools and knowledge, while wildlife management translates these advances into practical strategies aimed at biodiversity conservation and the maintenance of ecosystem health.

**Molecular Perspectives on Species Diversity, Conservation and Evolution:** A notable advance in biological research lies in the application of genetic approaches to unravel species diversity and evolutionary history. Four papers included in this issue demonstrate the power of complete mitochondrial genome sequencing (mitogenomics) as an essential tool for taxonomy and phylogenetics. Detailed studies on the genetics of crickets (genus *Ocellarnaca*) [1], a red-legged tarantula (*Brachypelma albiceps*) [2] and frogs (*Polypedates braueri* [3] and *Microhyla fissipes* [4]) not only resolve taxonomic ambiguities and refine phylogenetic relationships but also provide crucial molecular markers for conservation. In addition, genetic data are shown to support the forensic identification of illegally traded specimens [2].

**Population Dynamics and Habitat-Based Management:** Effective wildlife management relies on understanding population dynamics and species–habitat interactions. Three papers included in this issue provide long-term ecological or genetic data with direct implications for management practices. The results of a study on the migratory fish *Coilia nasus* [5] reveal pronounced sexual dimorphism and size-dependent migration, in which larger and more mature individuals move upstream. These findings are vital for evaluating the ongoing fishing moratorium and underscore the need to protect specific spawning habitats. In terrestrial ecosystems, research on rodent community structure in wetlands adjacent to a lake [6], using long-term time series and remote sensing-based vegetation index, establishes a clear relationship between vegetation cover and species density and fatness. This work provides a foundation for habitat management, which is crucial for preventing rodent population outbreaks and maintaining ecological balance. Complementarily, genetic analyses of an isolated population of the alpine salamander (*Salamandra atra*) [7] demonstrate that peripheral populations, although vulnerable due to low gene flow, harbor rare genetic variants that are important for the species’ overall adaptive potential, reinforcing the conservation priority of these unique genetic units.

**Global Pressures and Ecological Processes:** This issue culminates in the analysis of global threats that require complex and integrative responses, addressed through four papers. Climate change, in particular, is examined in two distinct studies. Records of Southern Bluefin Tuna (*Thunnus maccoyii*) and Pacific Bluefin Tuna (*Thunnus orientalis*) in Brazilian waters [8], genetically confirmed, suggest a possible climate-driven range expansion, implying an urgent need to reassess international spatial management frameworks. At a finer scale, in a study on a critically endangered salamander species (*Echinotriton chinhaiensis*) [9], the authors quantify the physiological vulnerability of larvae to warming, identifying a temperature threshold that restricts growth and movement and warning of the impacts of extreme climatic events. Lastly, ecosystem health is examined from an ecotoxicological perspective and through trophic interactions. Critically, the finding of a review on heavy metal contamination in Amazonian parrots [10] position these birds as sentinel species. The study findings highlight the neurotoxic and reproductive impacts of pollutants derived from anthropogenic activities, such as mercury and lead, emphasizing that pollution is an underestimated threat requiring enhanced biomonitoring and stricter environmental governance, with consequences not only for wildlife but also for human health. At a continental scale, coexistence between jaguars (*Panthera onca*) and pumas (*Puma concolor*) across their geographic distribution [11] was shown to be facilitated by a combination of taxonomic segregation strategies (a stabilizing mechanism) and convergence in prey functional traits (an equalizing mechanism). In this context, the equilibrium of competitive interactions and the conservation of apex predators depend intrinsically on maintaining the taxonomic and functional diversity of their prey, a challenge that is intensified in increasingly reduced and isolated environments.

The 11 papers featured in this Special Issue provide a representative overview of contemporary research in wildlife science. Together, they demonstrate that advances in wildlife conservation are closely linked to progress in biological research and adaptive approaches to biodiversity management. By addressing topics ranging from the resolution of taxonomic uncertainties through genetic approaches to the modeling of physiological responses to climate change and the evaluation of ecotoxicological threats, this collection offers an integrated perspective on key scientific and management issues. In this context, the combination of high-resolution analytical methods with long-term field-based ecology emerges as a critical pathway for developing conservation strategies that are not only responsive but also forward-looking and predictive. I invite readers to explore the breadth and diversity of these contributions, which collectively provide insights into ongoing challenges, emerging perspectives and future directions in wildlife conservation, management and biological research.
